# Bacterial Extracellular Vesicles at the Crossroads of Immune Regulation and Biofilm Dynamics: Biogenesis, Comparative Analysis, and Translational Challenges

**DOI:** 10.3390/biom16081132

**Published:** 2026-08-03

**Authors:** Qingyu Zhang, Beilei Zhang, Mohd Shafiq Aazmi, Lin Chen, Mohd Fakharul Zaman Raja Yahya

**Affiliations:** 1 Faculty of Applied Science, Universiti Teknologi MARA Shah Alam, Shah Alam 40450, Selangor, Malaysia; 2 Department of Food Research, Zhejiang Business College, Hangzhou 310059, China; 3 Sanmen Food and Drug Inspection and Testing Center, Taizhou 317100, China; 4 Integrative Pharmacogenomics Institute (iPROMISE), Universiti Teknologi MARA Selangor Branch, Puncak Alam Campus, Bandar Puncak Alam 42300, Selangor, Malaysia

**Keywords:** bacterial extracellular vesicles, membrane biogenesis, immune regulation, biofilm dynamics, probiotics, pathogenic bacteria

## Abstract

Bacterial extracellular vesicles (BEVs) are nano-sized lipid bilayer particles secreted by bacteria, capable of carrying various proteins, lipids, nucleic acids, and pathogen-associated molecular patterns (PAMPs). The biosynthetic pathway of BEVs determines their load components, physicochemical properties, and different biological activities. Increasing evidence indicates that BEVs play an important role in mediating host immune responses and the dynamic regulation of bacterial biofilms. Additionally, BEVs may serve as a molecular bridge between the two. BEVs derived from pathogens can trigger pro-inflammatory cascades, assist bacteria in immune evasion, and further accelerate the maturation of biofilms, forming a vicious cycle of persistent infection and inflammatory damage. In contrast, BEVs derived from probiotics can maintain host immune homeostasis and exert direct anti-biofilm and synergistic antibacterial effects, thereby breaking the pathological cycle. However, significant methodological research bottlenecks have greatly hindered the comparability and clinical translation of BEVs research. This article systematically summarizes the classification of BEVs and their biosynthetic mechanisms, compares the differential effects of BEVs from pathogenic bacteria and probiotic bacteria on immunity, clarifies the dual regulatory role of BEVs throughout the life cycle of biofilms, and highlights the bridging function of BEVs in the immune–biofilm interaction. Additionally, this article also discusses the current development of BEVs in clinical translation applications, such as vaccine development, antibiotic delivery, and mucosal inflammation intervention, and outlines the key industrial and clinical challenges faced in the future development of BEVs-based therapeutic approaches.

## 1. Introduction

### 1.1. Background and Research Objectives

Extracellular vesicles (EVs) are multiple nanoscale membrane vesicles released by cells (including eukaryotic cells, archaea, and bacteria), and these membrane vesicles are generally composed of a single-layer phospholipid bilayer-enclosed sphere with an inner lumen [1,2]. EVs are distributed in three fields of life, indicating EVs secretion is a common phenomenon of evolutionary conservation [1,3].

Bacterial extracellular vesicles (BEVs) are EVs secreted by various bacteria. The production of BEVs is a common phenomenon in bacteria. The formation of these vesicles is not only closely related to the physiological state of the bacteria, but is also influenced by environmental factors, such as nutritional conditions, stress states, and growth stages [4,5,6]. BEVs can carry various substances, including toxic factors, adhesins, DNA, RNA, communication compounds, toxins, immune regulatory factors, and nutrients. These substances are related to various biological functions such as toxicity, signal transmission, immune regulation, and biofilm formation [5,7,8]. Due to the unique structural and functional characteristics of BEVs, they show great potential in vaccine development, drug delivery systems, and diagnostic tools [9,10,11]. Currently, BEVs have become a hot area in biomedical research.

BEVs possess multiple biological functions, and the functions of BEVs from different sources vary. The immunomodulatory effect of BEVs on the host is currently a research hotspot, and the immunomodulatory effects of BEVs from different sources show significant differences. Moreover, studies have shown that BEVs are important regulatory factors for bacterial biofilm formation and inhibition [12]. Since BEVs play important roles in both immunity and biofilm formation, exploring the regulatory effects of BEVs in immunity and biofilm formation is helpful for understanding the role of BEVs in infection, inflammation and other disease processes. Furthermore, analyzing the bridging role of BEVs between immunity and biofilm is conducive to the development of new antibacterial strategies against drug-resistant bacteria based on BEVs.

However, most existing reviews discuss the immunomodulatory effects of BEVs and their regulatory role in biofilms separately, lacking a systematic analysis of the bidirectional interaction between immune regulation and biofilm dynamics, as well as the core bridging role of BEVs in this process. Meanwhile, the functional effects of BEVs remain controversial in current studies: BEVs can both promote and inhibit biofilm formation, and can both induce inflammatory responses and mediate immune tolerance. The final functional outcome is affected by multiple factors, such as strain type, dose and microenvironment.

This review systematically explores the biological functions and translational applications of BEVs, with a focus on their immunomodulatory effects, dynamic interplay with bacterial biofilms, in vivo fate, and the critical bridging role between immunomodulation and biofilm dynamics.

### 1.2. Literature Search Strategy and Scope

This paper is a structured narrative review. The literature was mainly retrieved from three databases: PubMed, Web of Science, Scopus and CNKI. The core search terms mainly include “bacterial extracellular vesicles”, “outer membrane vesicles”, “immune regulation”, “biofilm”, “pathogenic bacteria”, and “probiotics”.

This review focuses on studies published in recent 10 years, while classic foundational studies are not limited by publication time. Original research articles and systematic reviews with complete experimental data and clear conclusions were included, while meeting abstracts and case reports were excluded.

## 2. Biogenesis and Methodological Considerations of BEVs

### 2.1. Classification and Biogenesis Mechanisms of BEVs

Bacterial extracellular vesicles are nano-sized lipid bilayer vesicles secreted by bacteria, with a particle diameter ranging from 20 to 400 nm. The secretion of BEVs is a ubiquitous phenomenon that occurs throughout the entire bacterial life cycle [13]. The structure and function of BEVs are also different according to the type of source bacteria. Gram-negative bacteria produce BEVs more readily due to their unique cell envelope architecture, which consists of a thin peptidoglycan (PGN) layer and an outer membrane. Gram-negative BEVs typically contain outer-membrane proteins, lipopolysaccharides (LPS), and periplasmic contents [14].

According to the review published by Toyofuku et al. [1], BEVs include two major categories: B-type Membrane Vesicles (MVs) (generated by cell blebbing) and E-type MVs (formed by explosive cell lysis or bubbling cell death). B-type MVs include outer-membrane vesicles (OMVs), outer–inner-MVs (OIMVs), and cytoplasmic membrane vesicles (CMVs), while E-type MVs include explosive CMVs (ECMVs), explosive outer MVs (EOMVs), and explosive outer–inner MVs (EOIMVs) (Table 1). The contents of each type of BEV are different. Due to the different source strains and production methods, there are various types of BEVs, and during the extraction process, the obtained BEVs may contain multiple types.

### 2.2. The Regulatory Effect of the Biogenesis Mechanism on the Composition and Functional Characteristics of BEVs

The various biogenesis methods of BEVs will have different effects on their membrane structure and components, thereby causing differences in the functional characteristics of BEVs [15].

#### 2.2.1. Regulation of Lipid Composition

The lipid composition of BEVs directly reflects their membrane origin and biological generation pathway, and simultaneously determines the membrane fluidity, rigidity and membrane fusion characteristics of the vesicles [15,16,17]. The source of the membrane determines the composition of the lipid basis. The OMVs produced by Gram-negative bacteria are mainly composed of phospholipids (phosphatidylethanolamine, phosphatidylglycerol) and lipopolysaccharides [15,16]. CMVs produced by Gram-positive bacteria are mainly composed of phospholipids, without lipopolysaccharides but containing specific components, such as lipopolysaccharide-associated proteins [18]. The generation pathway also regulates the lipid sorting pattern. Under normal physiological conditions, exo-membrane budding exhibits a lipid sorting effect, preferentially enriching lipids specific to the exo-membrane. In response to stress, explosive lysosomal vesicles result from extensive disruption of cell membrane integrity, leading to a more mixed lipid composition. The disruption of membrane integrity causes random lipid encapsulation, resulting in a decrease in the proportion of lipids specific to the exo-membrane and a significant increase in the proportion of lipids specific to the endo-membrane [15,19]. Different induction conditions can also reshape the lipid enrichment profile of OMVs, generating characteristic lipid composition patterns. For example, when 1% glycine is added to induce the OMVs secreted by *E. coli* Nissle 1917, the levels of ceramide and lysophosphatidylcholine are significantly increased, while the levels of Bis (monoacylglycero)phosphate are significantly decreased. The lipids such as triglycerides and sphingomyelins show irregular changes. Furthermore, the particle size of the induced OMVs increases, their dispersion improves, and the absolute value of the Zeta potential becomes higher, reflecting the disturbance of this induction condition on the biogenesis process of vesicles, and specifically reshaping the lipid profile and physicochemical properties of OMVs [20]. In addition, the growth pattern also significantly affects the lipid functional properties of vesicles. The BEVs produced by *Pseudomonas aeruginosa* in a biological membrane environment are rich in C16/C18 long-chain saturated fatty acids and phosphatidylglycerol, and their membrane rigidity is significantly higher than that of BEVs derived from planktonic bacteria. These vesicles can be utilized by recipient bacteria, effectively reducing the membrane fluidity of the recipient bacteria and enhancing membrane rigidity, thereby promoting the development of the biofilm. However, the specific mechanism of this process still needs to be further verified.

#### 2.2.2. The Decisive Role in Loading Cellular Contents

The mechanism of formation directly determines whether the vesicles contain cytoplasmic components and the abundance and types of cytoplasmic contents [15]. Normal outer-membrane budding-generated OMVs only encapsulate outer-membrane proteins and periplasmic proteins, without soluble cytoplasmic proteins. However, OIMVs, stress lysosomal-type vesicles, and autolysis-related vesicles, due to the formation process involving inner-membrane disturbances or cell lysis, can carry a large amount of cytoplasmic proteins, metabolic enzymes, and stress response proteins [15,19]. Peptidoglycan deficiency is a key mechanism that promotes the incorporation of cytoplasmic components into vesicles. When peptidoglycan synthesis is impaired (such as nlpI mutation, glycine inhibition), the permeability of the bacterial outer membrane and inner membrane significantly increases. Cytoplasmic components leak into the periplasmic space and are captured by budding OMVs. Furthermore, the weakening of the peptidoglycan layer causes the inner membrane to protrude outward, forming OIMVs, which directly encapsulate the cytoplasmic contents. Together, these two processes increase the abundance of cytoplasmic components in the vesicles [21]. The loading of metabolites and small molecules is also regulated by the underlying mechanism. Normal OMVs selectively load metabolites from the periplasm. Under stress conditions, the integrity of the cell membrane is compromised, and more cytoplasmic metabolites are incorporated into the vesicles through passive diffusion [15,19].

#### 2.2.3. Impact on Pathogen-Associated Molecular Patterns (PAMPs) Load

The composition and load of PAMPs on the surface and inside of vesicles are directly determined by the biological production pathway and form the molecular basis for the differences in immunological activity of these vesicles [22]. The core PAMPs of BEVs produced by Gram-negative bacteria are LPS. The LPS composition of normal OMVs is basically the same as that of the mother cell’s outer membrane. Stress or lysis-derived vesicles can present different LPS structural characteristics due to membrane structure reorganization, thereby altering the activation efficiency of the Toll-like receptor 4 (TLR4) pathway [15,22]. The main PAMP of Gram-positive bacterial vesicles is lipoteichoic acid (LTA). CMVs released through the cell wall perforation pathway can retain more LTA and membrane-associated proteins, while vesicles derived from autolysis may present different surface PAMP distributions due to damage to the cell wall structure [23]. Peptidoglycan fragments and lipoproteins are also important components of vesicle PAMPs. OMVs produced by normal outer-membrane budding preferentially enrich peptidoglycan fragments and outer-membrane lipoproteins in the cytosol. OIMVs and vesicles derived from explosive lysis may carry more peptidoglycan degradation products, cross-linked structures, and cytoplasmic lipoproteins due to extensive degradation of the peptidoglycan layer and disturbance of membrane integrity, further enriching the types of PAMPs and more efficiently activating intracellular immune pathways such as nucleotide-binding oligomerization domain 1/2 (NOD1/NOD2) [15,21].

#### 2.2.4. Regulation of DNA/RNA Incorporation

Nucleic acid incorporation is a prerequisite for BEVs-mediated horizontal gene transfer, and its efficiency and type are strictly dependent on the biogenesis pathway [19,21]. Under normal physiological conditions, outer-membrane budding-generated OMVs rarely contain cytoplasmic nucleic acids. Nucleic acid incorporation is mainly achieved through three pathways: increased membrane permeability induced by peptidoglycan deficiency, formation OIMVs, and stress-induced explosive cell lysis [15,21]. When peptidoglycan synthesis is impaired, cytoplasmic DNA leaks into the periplasmic space and is captured by OMVs. Furthermore, OIMVs can directly encapsulate cytoplasmic nucleic acids, significantly increasing the DNA load of the vesicle and the number of single vesicle plasmids (up to 13 times the normal level under glycine induction) [21]. The content of stress-induced lysis-type vesicle nucleic acids is much higher than that of normal OMVs, and they can carry various types of nucleic acids, such as chromosomal DNA, plasmid DNA, mRNA, and ribosomal RNA fragments, making them efficient carriers for horizontal gene transfer between bacteria [19]. The mechanism of RNA incorporation is similar to that of DNA, and there may be a selective sorting process mediated by RNA-binding proteins. Stress conditions can significantly increase the total load and variety of vesicle RNA [15,19].

#### 2.2.5. Regulation of Immune Function Potential

The inflammatory potential and immunoregulatory properties of BEVs produced by different biological pathways show significant differences [22]. Normal exo-membrane budding-generated OMVs mainly activate the TLR4 signaling pathway through surface LPS, inducing the production of pro-inflammatory cytokines such as interleukin-6 (IL-6) and tumor necrosis factor-α (TNF-α), and participating in the host’s anti-infection immunity [22]. In contrast, stress-induced lysis-type vesicles, due to their rich nucleic acid components, such as chromosomal DNA and plasmid DNA, can further activate intracellular nucleic acid recognition receptors such as TLR9, triggering a more extensive immune response. The cytoplasmic virulence factors and regulatory proteins carried by these vesicles also work synergistically to further determine the intensity and direction of the inflammatory response [19]. The inflammatory effect of Gram-positive bacterial vesicles is mediated by LTA, peptidoglycan fragments, and membrane-related proteins. Different biological pathways (normal budding/autolysis release) result in significant differences in the types, abundance, and distribution of PAMPs on the vesicle surface, directly determining the intensity of the local inflammatory response, and this difference is closely related to the disease association of strain typing [23,24]. Under physiological conditions, some BEVs derived from gut symbiotic bacteria can accumulate immune-regulatory molecules such as indole metabolites and mucin-binding proteins, playing a role in maintaining intestinal immune homeostasis. The functional differentiation between these vesicles and pro-inflammatory vesicles is closely related to the selection of the biogenesis mechanism—vesicle components produced by the normal budding pathway are more selective and have more prominent immunoregulatory activity, while vesicles released by stress or lysis pathways have stronger inflammatory potential due to the mixed components [25,26].

#### 2.2.6. Impact on the Integration Ability of Biofilm Matrix

BEVs are important structural and functional components of the extracellular matrix of biological membranes. Their ability to integrate into the matrix is determined by their own lipid composition and surface properties, which are all regulated by the biogenesis mechanism [25]. Bacterial vesicles produced within the biological membrane are rich in C16/C18 long-chain saturated fatty acids and have higher membrane rigidity, making them more capable of binding to extracellular polysaccharides, extracellular DNA and other matrix components through hydrophobic interactions, significantly enhancing the structural stability of the biological membrane network [27]. Conventional outer-membrane budding-generated OMVs, due to their lipid composition being highly homologous to the bacterial outer membrane, are more likely to integrate with the membrane-derived matrix components of Gram-negative bacterial biofilms, while vesicles derived from lysis carry more cytoplasmic components and nucleic acids, and play a more significant role in delivering genetic material and regulating population functions in the matrix [26,27]. In addition, BEVs from biological membranes can enhance the rigidity of the cell membrane of planktonic bacteria, reduce membrane fluidity, and promote their transformation into a biofilm state, participating in the dynamic construction and maintenance of the biofilm [27].

In summary, there are many types of BEVs with certain differences in functionality and composition. And there are also differences in the naming of BEVs in different studies. In order to facilitate the overall analysis of the functions and characteristics of BEVs, we will uniformly use the abbreviation BEVs to refer to all types of bacterial extracellular vesicles throughout the subsequent review.

### 2.3. Methodological Challenges in BEVs Research

Although BEVs have great therapeutic potential, there are numerous methodological limitations within the research field of BEVs. These limitations severely impede the comparability and reproducibility of different research results, and have become a key bottleneck for the transition of BEVs from the laboratory to clinical applications [28,29].

#### 2.3.1. Heterogeneity of Isolation and Purification Methods

The lack of standardization in the separation and purification methods of BEVs is one of the core challenges in this field [28,29]. Currently, the commonly used methods for the separation and purification of BEVs mainly include differential ultracentrifugation (dUC), density gradient centrifugation (DGC), size-exclusion chromatography (SEC), ultrafiltration, immunoprecipitation, polymer precipitation, etc. [29,30]. Each method has its own inherent advantages and limitations. The BEVs obtained by different methods vary significantly in terms of yield, purity, integrity, and biological activity [28,30]. For instance, dUC is the most commonly used method for the large-scale separation of BEVs, but its high-speed centrifugation process may damage the structural integrity of BEVs and lead to the co-precipitation of protein aggregates and bacterial fragments, thereby reducing the purity of the sample [31]. In contrast, SEC can better maintain the morphological integrity of BEVs and effectively separate soluble proteins, but its processing capacity is limited and it is not suitable for large-scale preparation [31]. DGC, although it can provide higher-purity BEVs, has complex operation, a long processing time, and a low recovery rate, and may introduce residual contamination from the gradient medium. Barbieri et al. found that the samples obtained using the dUC method showed higher total protein content, wider particle size distribution, and more obvious non-vacuolated substance contamination, while SEC could produce vesicles with uniform morphology, intact structure, and a higher particle–protein ratio [30]. Even for the same type of bacterial BEVs, the results obtained by different laboratories often vary greatly. This kind of result discrepancy, caused by the separation and purification methods, seriously hinders the universality and reproducibility of the research conclusions [29,32].

In addition to isolation methods, the inherent heterogeneity of different BEV categories further increases the complexity of research. There are fundamental differences among natural BEVs, engineered BEVs, and drug-loaded BEVs, with varying levels of safety, immunogenicity and biological effects, but these differences are often not systematically compared in current studies. Natural BEVs from different bacterial sources exhibit significant differences in composition and function [33,34,35]. For instance, BEVs derived from pathogenic bacteria typically carry virulence factors (such as the CagA, VacA and HtrA proteases of Helicobacter pylori), enabling them to directly participate in the pathogenic process [33], while BEVs from probiotic sources usually possess functions such as immune regulation and maintaining intestinal homeostasis [34,35]. Engineered BEVs refer to BEVs produced by genetically modified parental bacteria, or chemically modified after vesicle isolation, to confer specific functions [36]. Currently, there are three main surface engineering modification schemes for BEVs: surface modification using the cytolysin A (ClyA) fusion system, surface modification using the lipoprotein–OmpA system (Lpp-OmpA system), and modification using the Tag/Catcher system [36]. The vesicular structure of BEVs enables them to serve as drug carriers, capable of carrying drugs for combination therapy [9]. At present, systematic comparative studies of these types of BEVs are often lacking in the research, making it difficult to determine the advantages of different strategies for specific indications.

#### 2.3.2. Sample Contamination and Purity Control Issues

During the isolation of BEVs, contamination of the product remains a concern. In the extraction process, potential contaminants such as bacterial debris, soluble proteins, nucleic acids, or endotoxins may be present [33,37,38]. Bacteria generate substantial amounts of cellular debris and cell wall components during cultivation, centrifugation, and lysis—fragments that are similar in size to BEVs [37]. These non-vesicular impurities closely resemble BEVs in terms of their physical properties, making it difficult for conventional separation methods like dUC and ultrafiltration to completely remove them, and thus leading to significant sample contamination [33]. For example, during differential ultracentrifugation, bacterial debris co-sediments with BEVs, resulting in the final product being mixed with numerous non-target particles [38]. Although density gradient centrifugation can improve purity, it also suffers from reduced recovery rates and complex operational procedures [38].

In addition, BEV samples may contain a significant amount of soluble contaminants. Common contaminants primarily include soluble proteins and nucleic acids derived from the culture medium or secreted by bacteria, as well as free LPS/endotoxins [33,38]. These soluble substances represent important interfering factors in functional studies of BEVs. For example, co-isolated soluble proteins might be incorrectly attributed to cargo proteins of BEVs, leading to inaccurate interpretations of BEV proteomics and function [38]. Similarly, free nucleic acids could introduce false-positive signals in gene regulation studies. Moreover, for BEVs derived from Gram-negative bacteria, co-isolated free LPS may compromise the accuracy of results in immune-related experiments [38].

#### 2.3.3. Dilemma of Standardized Quantification

The lack of a standardized quantitative method for BEVs is another critical challenge. Existing quantitative techniques have their own advantages and disadvantages, and a method that can comprehensively and accurately reflect the number of biological active vesicles is lacking [39]. The most commonly used quantitative methods include: nanoparticle tracking analysis (NTA), which determines particle concentration and size distribution by measuring Brownian motion, but this method cannot distinguish between BEVs, protein aggregates, and other nanoparticles, and has a large error rate in heterogeneous samples [39]; dynamic light scattering (DLS), which is suitable for the rapid measurement of particle size distribution, but is biased towards large particles and is not suitable for samples with high dispersion [30]; and protein quantification (such as the bicinchoninic acid assay), which is used to describe the total protein content of BEVs, but suffers from interference, mainly from co-separated soluble proteins [40].

However, the results obtained by different quantitative methods almost do not have a stable correlation: some samples have a high particle count, but the total protein content is low, making it difficult to uniformly standardize the dosage in in vitro and in vivo experiments [41]. Moreover, the particle–protein ratios of BEVs prepared from different bacterial sources and different isolation methods vary greatly, further increasing the complexity of quantitative and dosage conversion [39,40]. Therefore, current research urgently needs to establish a comprehensive quantitative strategy centered on functional dosage, combined with multiple physical and chemical detection methods.

#### 2.3.4. Limitations of In Vivo Tracing and Biodistribution Analysis

Studying the biodistribution of BEV in the body is of great significance for understanding its pharmacokinetics and targeting properties. In recent years, the advancements in technologies such as fluorescence labeling, radioactive labeling, and positron emission tomography (PET) imaging have led to significant progress in the pharmacokinetic studies of nanoparticles in vivo [42,43].

Fluorescence labeling and optical imaging are commonly used methods for tracking BEVs [44]. This approach typically integrates lipophilic fluorescent dyes or fluorescent proteins into the BEVs membrane to achieve non-invasive observation of deep tissues. This technique offers several advantages, including ease of implementation, low cost, and high spatial resolution, making it particularly suitable for in vitro assays, tissue section analysis, and studies of cellular uptake mechanisms [44,45]. For example, Jang et al. labeled BEVs with Cy7 near-infrared fluorescent dye and discovered the distribution of BEVs in mice after intraperitoneal injection [46]. Morishita et al. injected two types of Cy7-labeled BEVs and observed the specific transport and absorption mechanisms of these BEVs in various organs [47]. However, this method has poor tissue-penetration ability and is susceptible to interference from spontaneous fluorescence, making it difficult for it to be used for precise quantitative analysis of deep organs.

PET imaging is currently the mainstream quantitative tracing method. Its core advantage lies in its high sensitivity, absolute quantification capability, and ability to conduct dynamic monitoring [43]. This method anchors the chelating agent at specific sites on the bacterial surface display system (such as SpyCatcher/SpyTag) to be fixed on the BEV membrane, and then combines with radioactive nuclides (such as ^64^Cu) [43]. Studies have shown that after the intravenous injection of ^64^Cu-labeled BEVs, rapid enrichment and retention of BEVs in the liver and spleen can be clearly observed at multiple time points, precisely revealing the distribution pattern dominated by the reticuloendothelial system (RES) [43]. This method overcomes the shortcomings of fluorescence labeling, which is prone to quenching and has a limited penetration depth, and is the mainstream method for studying the whole-body pharmacokinetics of BEVs. At present, most studies can only provide macroscopic distribution information of BEV at the organ level, while research at the cellular and subcellular levels has made slow progress.

## 3. Immunomodulatory Effects of BEVs

**Figure 1:**
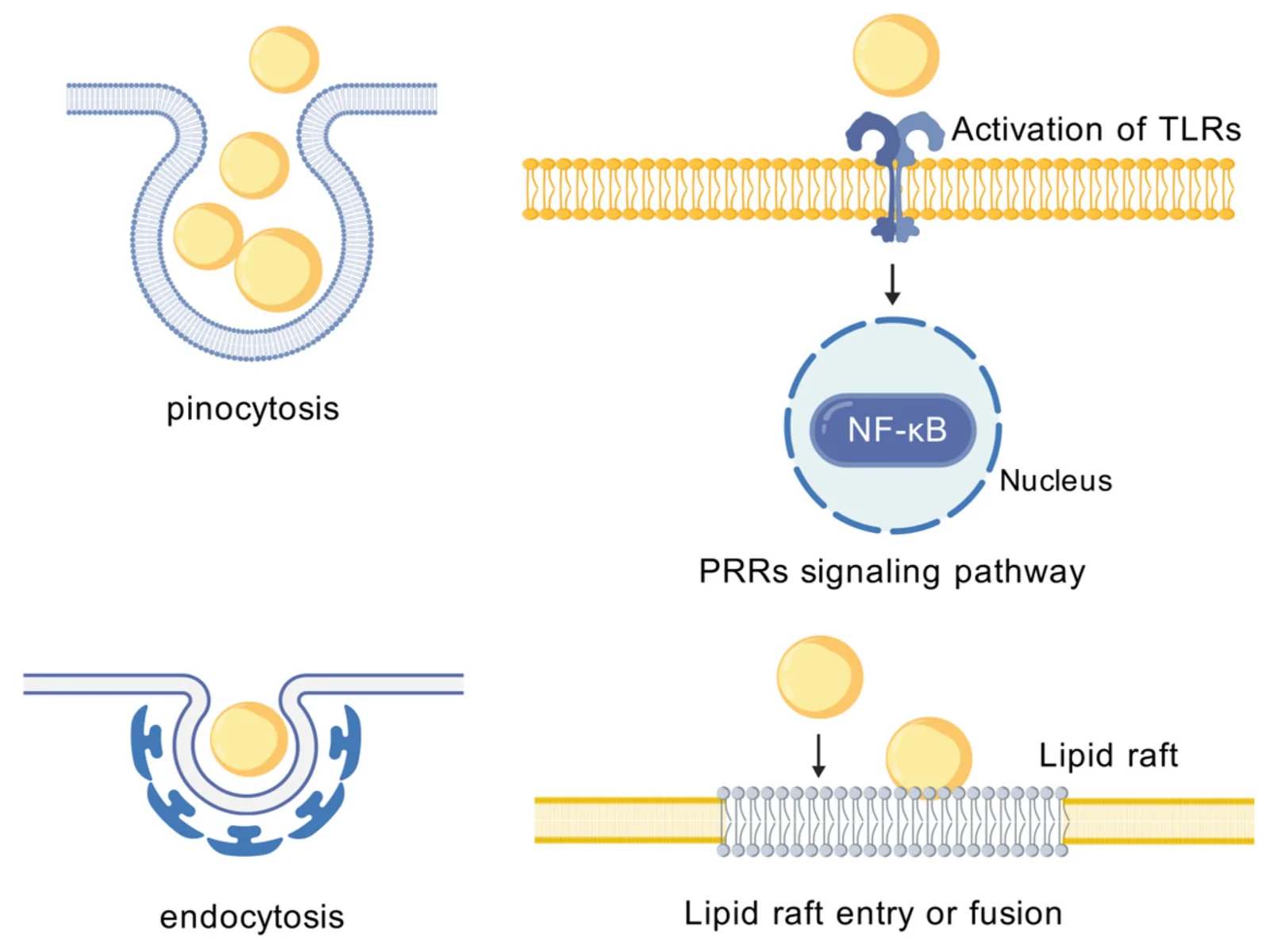
The mode of action of BEVs on host cells. Abbreviations: PRRs, pattern recognition receptors; TLRs, Toll-like receptors; NF-κB, nuclear factor kappa-B.

Studies have shown that pathogenic BEVs can transmit a variety of signaling molecules to host cells and cause immune responses. BEVs generally interact with host cells through four pathways (Figure 1): (1) ligation of cell surface pattern recognition receptors (PRRs); (2) fusion with lipid raft domains; (3) pinocytosis; and (4) clathrin- or caveolin-mediated endocytosis [1,48,49,50,51]. The PRRs signaling pathway is the main pathway through which BEVs induce host cell immune responses. The surface and interior of BEVs carry various microbe-associated molecular patterns/pathogen-associated molecular patterns (MAMPs/PAMPs), such as lipopolysaccharides and nucleic acids. After BEVs reach the host cell, these substances are recognized by PRRs in immune and non-immune cells, stimulating the host cell to produce a series of cytokines, and thereby activating the immune response [52]. When interacting with epithelial cells, pathogenic BEVs can activate inflammatory response through TLRs and nucleotide-binding oligomerization domain-like receptor (NLR)-mediated signaling pathways [51]. BEVs derived from some probiotic strains can also inhibit host cell inflammatory responses through the TLRs signaling pathway. Koh et al. [53] found that *Lactobacillus gasseri* BEVs inhibit macrophage RAW264.7 inflammatory response through the TLR4/nuclear factor kappa-light-chain-enhancer of the activated B cells (NF-κB) signaling pathway. BEVs help induce disease when bacteria infect the host, but when an appropriate amount of BEVs is injected into animals alone, they will induce a protective immune response to resist pathogen infection [54].

### 3.1. Immune Response Caused by Pathogenic BEVs

#### 3.1.1. Pro-Inflammatory Response and Immunopathological Damage

Gram-negative pathogens can cause host inflammatory response through BEVs [55]. These BEVs come into contact with host cells through adhesion or fusion, and stimulate host cells to produce immune responses by carrying virulence factors, thereby causing cellular inflammation [55,56]. Most BEVs from Gram-negative pathogens contain disease-related pathogenic molecules such as LPS, lipoproteins, and flagellin, which can interact with host cell receptors and trigger an inflammatory response [57]. For example, the BEVs secreted by *E. coli* (STEC), which produces Shiga toxin contain Shiga toxin 2a (Stx2a), cytolethal distending toxin V (CdtV), flagellin, and LPS [58]. The BEV secreted by *Helicobacter pylori* contains various proteins that can cause inflammation in epithelial cells [59]. *E. coli* BEVs can deliver toxins to host cells, induce the production of type I interferon via TLR4–TIR-domain-containing adaptor-inducing interferon-β (TRIF) signaling, activate caspase-11 non-classical inflammasome, trigger immune response and cell pyroptosis [60]. In addition, Gram-negative BEVs can selectively accumulate near tumor cells, and may change the tumor microenvironment and affect the subsequent development of tumor cells [61].

BEVs from Gram-positive pathogenic bacteria can also cause inflammatory reactions [62,63,64]. *Staphylococcus aureus* BEVs can induce the expression of pro-inflammatory and pyroptosis genes in bovine monocyte-derived macrophages [62]. *Mycobacterium tuberculosis* BEVs can activate the inflammatory response of host cells and inhibit the activation of T cells [63]. *Filifan alocis* BEVs can express pro-inflammatory cytokines in THP-1 cells and HOK-16B cells, and their immunostimulatory properties are similar to those of bacteria themselves [64].

#### 3.1.2. Immune Evasion and Immunosuppressive Effects

In addition to triggering inflammatory damage, pathogenic BEVs can assist bacteria in immune evasion. BEVs can act as “molecular decoys” to bind and inactivate harmful substances such as complement and antibodies, competing for the binding of immune effectors, weakening the immune factor effect, reducing the probability of the source bacteria being recognized, and protecting the source bacteria [65]. Studies have also confirmed that *S. mutans* BEVs can significantly reduce the phagocytic ability of macrophages against bacteria, creating conditions for bacterial colonization and persistent infection [66].

### 3.2. Immune Response Caused by Probiotic BEVs

However, probiotic BEVs carry little or limited virulence factors and will not induce disease and inflammation at physiological doses [67,68]. Studies have shown that BEVs from some intestinal probiotics can carry a variety of signaling molecules to communicate with host cells through the mucus layer, and enhance host immunity by participating in the regulation of different signal pathways and immune responses [69,70]. In addition, compared with probiotics themselves, EVs more easily spread, cross the mucus layer, and migrate directly to the intestinal submucosa to interact with different cells of the host immune system. Therefore, BEVs intake is a safe and effective way to provide the beneficial effects of probiotics, while avoiding the adverse effects of probiotics on some sensitive populations [71,72].

#### 3.2.1. Protective Immune Enhancement

Gram-negative probiotics (such as *B. fragilis, B. vulgatus*) can deliver immunomodulatory functional molecules to the host through BEVs and regulate the host immune response [14,69]. *E. coli* Nissle 1917 BEVs can activate intestinal mucosal immune and defense responses and enhance barrier function by increasing the expression of tight junction protein in intestinal epithelial cells [73,74].

The BEVs secreted by Gram-positive probiotic can induce host cells to produce cytokines, inhibit inflammatory response, activate defense genes and promote intestinal IgA secretion [68,75,76]. Probiotic BEVs can play an immune-protective role by activating host defense genes. Li et al. [77] studied the role of BEVs from *Lactiplantibacillus plantarum* in the treatment of drug-resistant pathogens and found that it significantly increased the transcription of defense genes CPR-1 and CLEC-60, upregulated the expression of host defense genes and protected the host. In addition, some probiotic BEVs can promote intestinal immunoglobulin A (IgA) secretion [78]. For example, BEVs secreted by *Lactobacillus sakei* subsp. *sakei* NBRC15893 subspecies can activate TLR2 (Toll-like receptor) signal through its cell wall components and promote the production of IgA by murine Peyer’s patch cells, which is a new role between intestinal microorganisms and the host through BEVs [79]. Such immune enhancement makes BEVs extracted from some probiotic strains available as a safe adjuvant [79].

#### 3.2.2. Anti-Inflammation and Immune Tolerance Regulation

At present, the research on Gram-negative probiotic BEVs mainly focuses on three symbiotic bacteria: *B. fragilis, B. vulgatus* and *E. coli* Nissle 1917. Shen et al. [80] found that *B. fragilis* can release PSA through BEVs, induce immune regulation, induce and enhance regulatory T cells (Tregs) function, increase interleukin-10 (IL-10) levels, and treat experimental colitis in mice. Further studies found that *B. fragilis* BEVs-mediated immune regulation was related to autophagy genes in the non-standard autophagy pathway [81]. Maerz et al. [82] found that *B. vulgatus* BEVs exert anti-inflammatory effects by inducing tolerogenic CD11c^+^ dendritic cells in the colonic lamina propria. *E. coli* Nissle 1917 BEVs can reduce the level of proinflammatory factors, reduce the activity of inflammatory enzymes (such as cyclooxygenase 2), enhance the expression of intestinal barrier markers, and then inhibit the development of dextran sulfate sodium (DSS)-induced colitis in mice [83].

BEVs from Gram-positive probiotics also have significant anti-inflammatory activity. For example, BEVs from *Lactobacillus paracasei* can reduce the expression of LPS-induced inflammatory cytokines (IL-1α, IL-1β, IL-2 and TNFα) and increase the production of anti-inflammatory cytokines IL-10 and TGFβ [84]. *L. paracasei* BEVs can regulate TNFα, reduce inflammatory response, and improve inflammation-induced skin phenotype [85]. Notably, some of these beneficial probiotic effects are more pronounced or only observed with BEV administration when compared to treatment with intact probiotic bacteria [86].

The immunomodulatory effects of BEVs are not limited to directly altering the host immune state. Changes in the local immune microenvironment will further affect bacterial colonization efficiency and biofilm development. BEVs may act as a shared mediator, potentially forming a closely linked functional loop between immune regulation and biofilm dynamics.

## 4. Relationship Between BEVs and Biofilm

**Figure 2:**
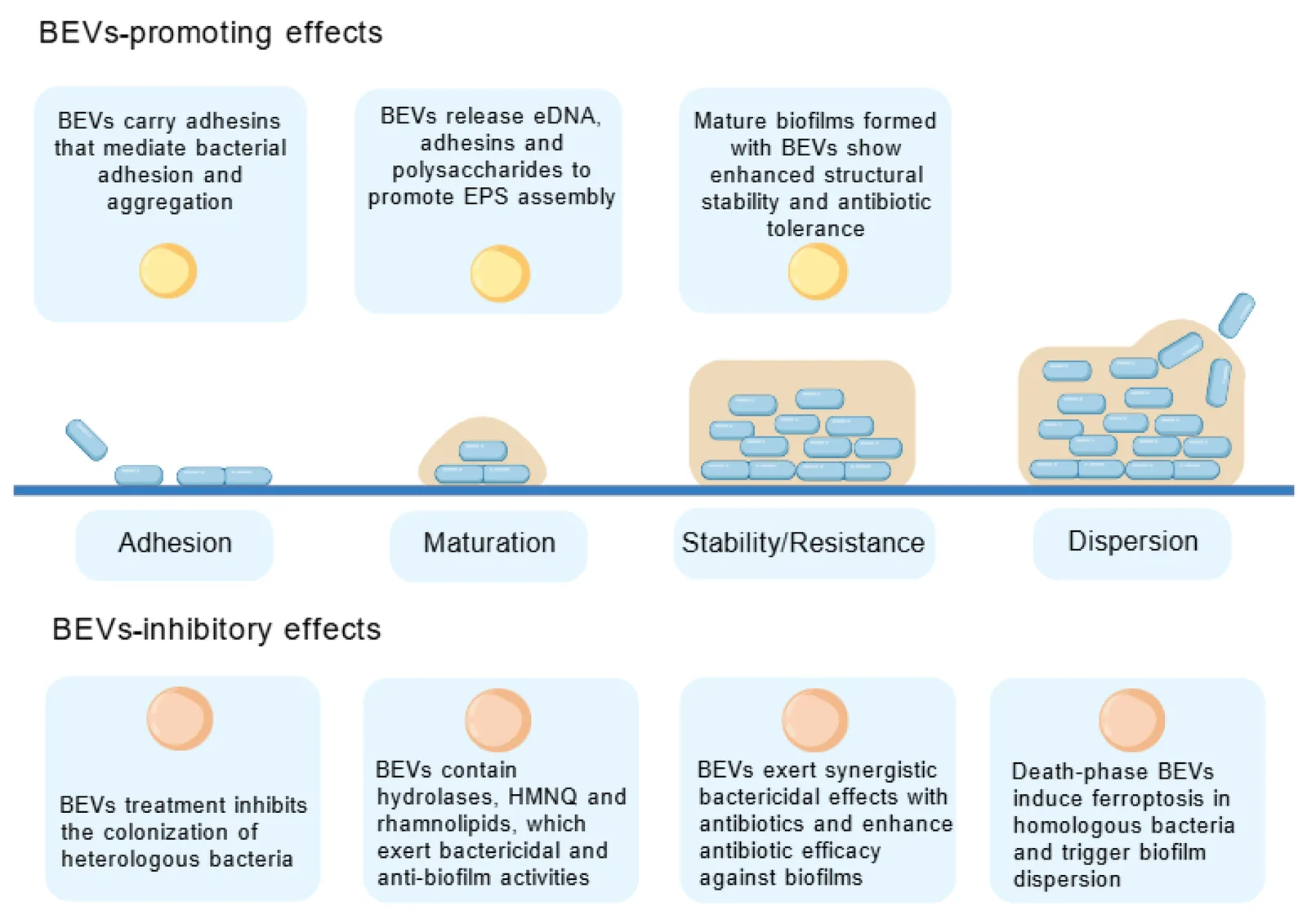
The formation process of biofilm and the regulatory effect of BEVs on biofilm. Abbreviations: BEVs, bacterial extracellular vesicles; EPS, extracellular polymeric substances; eDNA, extracellular DNA; HMNQ, 4-hydroxy-3-methyl-2-(2-non-enyl)-quinoline; QS, quorum sensing.

Biofilms are structured, complex three-dimensional communities of microorganisms embedded in a self-produced extracellular matrix [87]. Biofilms typically adhere to biotic or abiotic surfaces and are enclosed within an extracellular polymeric substance (EPS) matrix secreted by the resident microorganisms [88,89]. Biofilm formation is a lifestyle adopted by most microorganisms in nature [90], and the vast majority of bacteria are capable of forming biofilms under appropriate conditions. The structure of the biofilm is complex and its internal distribution is uneven. The structure of the biofilm is closely related to the bacterial species and the environmental conditions of the strain. Even in the same biofilm, different bacterial individuals show heterogeneity in their gene expression and physiological activity because they develop at different times and different spaces in the biofilm [91]. Biofilm has a barrier-protective effect on bacteria, which can increase the resistance of pathogenic bacteria to antibiotics by 10–1000 times [92]. In addition, biofilm can help pathogenic bacteria avoid host immune attack and improve the stress resistance of pathogenic bacteria under extreme conditions [93,94]. Biofilm formation by bacteria is a complex dynamic process, which can be generally divided into five stages: (1) reversible attachment stage; (2) irreversible attachment stage; (3) microcolony formation stage; (4) biofilm maturation stage; (5) bacterial abscission/diffusion stage (Figure 2) [91,95].

Biofilm is mainly composed of bacteria and EPS components secreted by bacteria. Based on their binding affinity to bacterial cells, EPS can be operationally divided into two fractions: tightly bound EPS (TB-EPS), which is closely associated with the cell surface, and loosely bound EPS (LB-EPS), which is dispersed in the intercellular space [96]. Generally, EPS is mainly composed of water (97%), extracellular polysaccharides, extracellular proteins, extracellular DNA (eDNA) and lipids [97,98]. The composition of EPS varies according to the bacterial species, micro ecological environment and nutritional conditions. Extracellular polysaccharides endow bacteria with adhesion characteristics to EPS and provide shape and structural support for biofilm [99]. Extracellular proteins play the role of an “adhesive polymer” and form gelatinous regions, thus forming a microenvironment around the cells [100]. eDNA carries negative charge and interacts with basal surface receptors to promote microbial adhesion [101]. This eDNA is also involved in regulating the formation process and maintaining the spatial stability of biofilms. The positively charged exopolysaccharide Pel in *Pseudomonas aeruginosa* biofilms can cross-link with extracellular eDNA via ionic interactions, thereby providing structural integrity to the biofilm matrix [102].

### 4.1. The Differences in Biofilms Formed by Gram-Positive Bacteria and Gram-Negative Bacteria

Due to the differences in cell structure, the ways in which Gram-negative and Gram-positive bacteria form biofilms also differ.

#### 4.1.1. Gram-Negative Bacteria

Gram-negative bacteria rely on LPS for adhesion due to their unique outer-membrane structure [103]. During biofilm formation, Gram-negative bacteria mainly secrete BEVs through outer-membrane blebbing, and these BEVs participate in multiple processes of biofilm development. BEVs carry outer-membrane proteins, LPS and periplasmic components, which can not only mediate initial bacterial adhesion, but also provide structural components and signaling molecules for the biofilm matrix [103,104].

In terms of mechanical properties, current research indicates that the overall viscoelasticity and rigidity of Gram-negative bacterial biofilms are generally lower than those of Gram-positive bacteria [105,106,107]. In vitro experiments revealed that the storage modulus of the interface membrane of *Staphylococcus aureus* (Gram-positive) was approximately 30–38 mN/m, while that of the wild-type *Pseudomonas aeruginosa* (Gram-negative) was about 41 mN/m, and that of the mucoid type was approximately 37 mN/m. Although there were fluctuations at the single-strain level, the overall trend shows that Gram-negative bacteria, due to the presence of the outer membrane, reduce the direct cross-linking between the cell wall and the extracellular matrix. Moreover, their own matrix polysaccharides (such as alginate) tend to form a looser network structure, resulting in a lower cross-linking density and weaker mechanical stability, and making them more prone to deformation [106,107].

#### 4.1.2. Gram-Positive Bacteria

Gram-positive bacteria have no outer membrane, and their cell walls contain sugar polymers, such as Teichoic Acids (TAs). These TAs mediate adhesion by influencing surface charge and hydrophobicity [103]. Gram-positive bacteria produce BEVs mainly through cytoplasmic membrane blebbing, and the vesicles carry more cytoplasmic proteins, DNA and RNA, which participate in biofilm construction and intercellular communication.

Current evidence suggests that Gram-positive bacterial biofilms generally exhibit higher rigidity and viscoelasticity than Gram-negative ones, primarily due to differences in the cell-envelope architecture: Gram-positive bacteria possess a thick peptidoglycan layer (20–80 nm) without an outer membrane, whereas Gram-negative bacteria have a thin peptidoglycan layer (3–8 nm) shielded by an outer membrane. It is widely hypothesized that the thick peptidoglycan provides abundant anchoring sites for matrix components, forming a more highly cross-linked three-dimensional network, though this specific mechanism remains to be directly verified in these studies [106,107]. Studies have confirmed that the viscoelastic ratio of *Staphylococcus aureus* colonies is 1.77 times that of Gram-negative bacteria such as *Acinetobacter baumannii* [105].

#### 4.1.3. *Candida* spp.

In addition to bacteria, *Candida albicans* can also secrete extracellular vesicles during biofilm formation. The composition of biofilm-derived vesicles of *Candida albicans* is very similar to that of biofilm matrix materials. These vesicles play a key role in the production of EPS and biofilm drug resistance, and are an important functional component of the fungal biofilm matrix [108].

### 4.2. The Role of BEVs in the Process of Biofilm Formation

#### 4.2.1. Initial Adhesion and Microcolony Formation Stage

During the initial stage of biofilm formation, BEVs act as surface modification tools, adhesion mediators and matrix nucleation cores, and are key regulatory factors for the surface colonization and initiation of biofilm assembly by planktonic bacteria [109,110]. Under external stress, some Gram-negative bacteria can secrete large amounts of BEVs, which significantly increase the hydrophobicity of the bacterial surface, thereby promoting the initial adhesion of bacteria to solid surfaces and accelerating the early formation of biofilms. For example, after *Pseudomonas putida* is subjected to alcohol stress, the amount of BEVs increases significantly, and the hydrophobicity of the bacterial biofilm significantly improves [109]. This change in hydrophobicity directly enhances the binding ability of bacteria to solid substrates and promotes the formation of biofilms.

In addition, BEVs themselves are the core components of EPS and promote adhesion at the initial stage of biofilm formation. The lipid proteins and surface antigens on the vesicle surface can mediate the initial interaction between bacteria and the substrate, making planktonic cells more likely to aggregate into microcolonies. For instance, the BEVs secreted by *Porphyromonas gingivalis* contain adhesins, which contribute to the development of plaque biofilms [110]. Furthermore, BEVs can serve as a nucleation site for eDNA by packaging and releasing key macromolecules such as EPS components, extracellular polysaccharides, lipids, and functional proteins, providing anchor points for the aggregation of planktonic cells and promoting the rapid formation of microcolonies [3,111]. Studies have confirmed that BEVs can promote biofilm formation by providing beneficial functional proteins such as heme-utilizing protein HmuY [3], and BEVs themselves are also structural units of biofilms and key materials for constructing and stabilizing an early solid structure for biofilms [65]. Moreover, BEVs can directly promote the early formation of biofilms by secreting EPS components, transmitting membrane-related functional genes, and directly promoting the formation of biofilms [111,112,113]. For example, the BEVs of *P. gingivalis* carry virulence factors such as gingivin, which can regulate bacterial aggregation behavior and enhance the stability of biofilms [12].

Some studies have found that BEVs exhibit competitive inhibitory effects during the initial stage of biofilm formation. During the initial stage of biofilm formation, in addition to promoting the colonization of the bacteria and the symbiotic bacteria themselves, some strains of BEVs can specifically inhibit the initial formation of biofilms by both their own and other bacterial species [114]. BEVs derived from *Burkholderia thailandensis* can also be used as an aggressive tool by certain bacteria to inhibit and kill other competitive bacteria, as well as to disrupt their biofilms [8].

#### 4.2.2. Maturation and Stability Maintenance Stage

During the mature stage of the biofilm, BEVs act as the core transport carriers of the extracellular components of bacteria, maintaining the stability of the biofilm through means such as structural reinforcement, nutrient sharing, gene exchange and drug-resistance transmission [108,115,116]. BEVs are the main carriers for transporting structural components into the biofilm matrix, and they can promote the synthesis and deposition of EPS matrix in a dose-dependent manner, strengthening the three-dimensional network structure of the biofilm. Studies have shown that the exogenous addition of purified BEVs can significantly promote the formation of biofilms, and the amount of biofilm formation increases significantly with the increase in the amount of added BEVs, showing a clear dose-dependent relationship. Moreover, the proteins carried by BEVs are the key substances for strengthening the biofilm matrix network [115]. Proteomics analysis has confirmed that approximately 20% of the protein groups in the matrix of mature biofilms of *P. aeruginosa* come from BEVs-related proteins [116].

BEVs are the core mediators for intraspecific and interspecific communication among bacteria within mature biofilms [117,118]. They can precisely regulate the collective behavior of the bacterial community by transmitting quorum sensing (QS) signal molecules and regulatory proteins, thereby promoting the maturation and maintaining the stability of the biofilm. BEVs help bacteria in biofilm transmit signals to each other, and can mediate intercellular communication through QS signal transduction molecules to promote biofilm formation and maturation [117]. Studies have shown that BEVs, as a new type of intraspecific communication tool, precisely regulate group sensing and group behavior by transmitting protein signaling molecules ObfA. For example, *Vibrio cholerae* BEVs derived from biofilms contain the outer-membrane protein ObfA. This protein inhibits the activity of HapR (a key regulatory factor for group sensing), thereby enhancing biofilm formation [118].

BEVs can act as “public goods”, enabling the sharing and delivery of nutrients among species, and supporting the maintenance of the homeostasis of biofilm matrix in resource-poor environments [119,120]. Research has found that the BEVs released by *Dietzia sp.* DQ12-45-1b serve as a crucial extracellular “public good”. By packaging and delivering specific metabolites (iron carriers and antioxidant molecules), they share essential nutrients (iron) among homologous species, thereby supporting the adaptation and survival of microbial communities in resource-poor environments [119]. BEVs can deliver nutrients to the biofilm and help bind or inactivate harmful substances such as antibiotics, a complement system (a core humoral component of innate immunity) and antibodies [120].

The composition and function of BEVs within biofilms exhibit significant heterogeneity, which is dependent on the bacterial species, growth conditions, and substrate environment [4,121]. The characteristics of BEVs from *Streptococcus mutans* are influenced by both the bacterial growth mode (free-floating or biofilm) and the substrate environment, potentially enhancing the pathogenicity of the bacteria [121]. When *S. mutans* forms biofilms on collagen matrices, the production, size, and protein composition of its secreted BEVs undergo significant changes, and the glycation of collagen (simulating aging or a high-glucose environment) further regulates this process [121]. Furthermore, the environmental factors within the biofilm matrix (such as pH and oxygen tension) further regulate the release of BEVs and cargo selection, optimizing the survival strategies of the bacterial population [121,122]. Oral pathogenic bacteria can secrete BEVs of the bacteria that carry multiple virulence factors. The generation and release of these BEVs are also dynamically regulated by the physicochemical factors in the biofilm microenvironment (such as local pH reduction, changes in oxygen tension, etc.). This environment-dependent mechanism of BEV release enables bacterial populations to flexibly adjust their virulence expression and group behavior in response to changes in the oral microecology, thereby optimizing their adaptability and pathogenic potential in complex oral environments [122].

#### 4.2.3. Dispersion Stage

During the dispersion stage of the biofilm, some bacteria within the mature biofilm degrade the EPS matrix and detach from the community, entering a planktonic state, and then spread to new environments to colonize and initiate a new round of biofilm formation. During this process, BEVs may mediate matrix dissociation and community remodeling through the enzymes they carry. Studies have found that many bacteria can secrete BEVs containing abundant lysozymes. For example, *Lysobacter gummosus* can secrete BEVs containing lysozymes, and these vesicles not only lyse various Gram-positive bacteria, but can even have a strong lytic effect on *P. aeruginosa* and other bacteria [123]. Such BEVs containing special enzymes may play a role in the dispersion of biofilms, but there are currently no studies on BEVs mediating biofilm disintegration.

### 4.3. Sources of Variability in BEVs-Mediated Biofilm Modulation

While many studies support a role for BEVs in promoting biofilm formation and stability, the literature also contains significant discrepancies that warrant critical discussion. Notably, Baeza and Mercade [124] reported that among 11 cold-adapted Gram-negative bacterial species, BEV production showed no correlation with biofilm-forming capacity in 10 species, with only *Shewanella vesiculosa* M7 exhibiting enhanced biofilm formation via eATP-containing BEVs. This finding challenges the generality of BEV-mediated biofilm promotion and highlights the need to consider multiple confounding variables.

BEVs production and cargo selection are dynamically regulated by environmental factors including nutrient availability, oxygen tension, pH, and growth phase. BEVs harvested at different growth stages (exponential, stationary, death phase) can have opposing effects on biofilms. For example, *P. aeruginosa* BEVs from the death phase inhibit biofilm formation via iron-dependent mechanisms, while BEVs from earlier stages can promote biofilm development [113].

Substrate type and biofilm maturity may also influence BEV effects. The effect of BEVs may depend on whether they act during initial attachment, microcolony formation, or mature biofilm stages. During the initial stage of biofilm formation, BEVs act as surface modification tools, adhesion mediators and matrix nucleation cores, and are key regulatory factors for the surface colonization and initiation of biofilm assembly by planktonic bacteria [109,110]. BEVs may promote initial adhesion but have different effects on pre-formed biofilms.

Differences in bacterial species and envelope structure may contribute to the observed variability in BEV effects on biofilms. Gram-negative and Gram-positive bacteria produce fundamentally different types of BEVs with distinct membrane compositions and cargo profiles, which likely result in different biofilm-modulating activities [103]. Even within Gram-negative species, variations in outer-membrane composition and vesicle biogenesis mechanisms can lead to divergent effects on biofilms [103]. However, systematic studies comparing BEV functions across diverse bacterial species and envelope types remain scarce, and further research is needed to clarify these potential relationships.

Conceptual confusion between biofilm inhibition, disruption, dispersal, and bacterial killing may also contribute to the inconsistent findings. Many studies report “anti-biofilm activity” without clearly distinguishing between inhibition of formation, disruption of pre-formed biofilms, induction of dispersal, or bactericidal effects on biofilm-associated bacteria. This lack of terminological precision makes direct comparison between studies difficult.

Taken together, the effect of BEVs on biofilms is highly context-dependent and cannot be generalized across species, conditions, or experimental systems.

### 4.4. Antibacterial and Anti-Biofilm Properties of Heterologous BEVs

Some heterologous BEVs have antibacterial and antibiofilm potential, even including a bactericidal effect on drug-resistant bacteria. Li et al. [125] found that BEVs from 15 strains of bacteria, such as *Escherichia, Klebsiella, Morganella* and so on, have bactericidal effect on 17 strains of bacteria, which include Gram-negative bacteria, Gram-positive bacteria and several representative drug-resistant pathogens. However, the bactericidal ability is seemingly selective. In addition, all of these BEVs form 15 strains of bacteria that have no broad-spectrum bactericidal ability. Furthermore, some probiotic BEVs possess anti-biofilm properties. The BEVs of *Lpb. plantarum* can inhibit the biofilm of *L. monocytogenes* [126].

BEVs contain many hydrolases which have a bactericidal effect on microorganisms. Peptidoglycan hydrolase is one of the respective hydrolases in BEVs, which can hydrolyze peptidoglycan in the cell wall and cause cell lysis. For Gram-positive bacteria, BEVs can adhere to the cell wall, release hydrolase to the adhesion point, and hydrolyze peptidoglycan, resulting in rupture of cell wall at the adhesion point and cell death. For Gram-negative bacteria, due to the protective effect of the outer membrane on peptidoglycan, free peptidoglycan hydrolase generally struggles to kill bacteria. BEVs can fuse with the outer membrane and deliver peptidoglycan hydrolase into periplasmic space, hydrolyze peptidoglycan at multiple sites, and cause cell death [125]. In the process of BEVs killing bacteria, the fusion of EVs with bacterium is very important. Evans et al. [127] found that the addition of fusogenic enzyme can enhance the bactericidal ability of BEVs from *Myxococcus xanthus* to *E.coli*, indicating that the fusion of EVs with bacteria is an important process by which EVs can play an antibacterial role. The bactericidal ability of peptidoglycan hydrolase contained in EVs is related to the type of peptidoglycan in the target cell. If the type of peptidoglycan in the target cell is similar to the bacteria that BEVs come from, EVs seemingly can effectively kill the target bacteria [125]. In addition to peptidoglycan hydrolases, BEVs contain numerous other lethal cargo molecules that contribute to their antibacterial activity [128].

BEVs have synergistic effects with antibiotics and improve the bactericidal ability of antibiotics [129,130,131]. It should be noted that this phenomenon is an interspecific competition mechanism of bacteria: under antibiotic stress, bacteria produce antibiotic-loaded BEVs that mainly kill heterologous competing bacteria, which is different from the drug-resistance mechanism through which BEVs in pathogenic biofilms adsorb antibiotics to protect their own flora.

Antibiotics can induce the production of BEVs, and these BEVs will contain antibiotics and have a strong antibacterial ability. Gentamicin can induce *P. aeruginosa* produce BEVs, which is threefold above the normal level of the formed BEVs [129]. These BEVs contain gentamicin and more autolysins, and also have strong bactericidal ability (which is 2.5 times that of gentamicin MIC) [130]. In addition, gentamicin-containing BEVs can penetrate eukaryotic cells and kill the intracellular pathogens [129]. The BEVs produced by *E. coli* Nissle 1917 can also carry tobramycin (TOB), demonstrating antibacterial and anti-biofilm capabilities. The killing effect of these drug-loaded BEVs on bacteria within biofilms is 20,000 times stronger than that of free TOB [131].

BEVs and its contents have anti-biofilm ability [132]. BEVs can inhibit the production of biofilms by other bacteria. After treating the surface of polystyrene with *Staphylococcus aureus* BEVs, the hydrophilicity can be increased and the biofilm formation of various pathogenic bacteria can be effectively inhibited [133]. Wang et al. [8] found that *Burkholderia thailandensis* BEVs contain 4-hydroxy-3-methyl-2-(2-nonenyl)-quinoline (HMNQ) and rhamnolipids, which have bactericidal and anti-biofilm properties. *Burkholderia thailandensis* BEVs, HMNQ and rhamnolipid can inhibit pre-formed biofilm, disrupt biofilm structure, reduce biofilm biomass, and kill the bacteria in the biofilm. Furthermore, both heat-labile and heat-stable components possess antibiofilm activity. Baker et al. [134] found that BEVs from *Burkholderia thailandensis* can inhibit biofilm formation by *P. aeruginosa*. Even from the same strain, BEVs contain different substances at different growth stages, which can have varying effects on biofilms. The *P. aeruginosa* BEVs produced at different growth stages have a promoting or inhibitory effect on biofilms. The BEVs of *P. aeruginosa* during the death period can regulate an iron-dependent mechanism (presumably ferroptosis) and inhibit biofilm formation [113].

### 4.5. The Difficulties Brought by BEVs to Anti-Biofilm Treatment

BEVs are not only the important regulatory factors for maintaining the structure of biofilms, but also an important factor leading to the failure of clinical treatment and repeated infections of biofilm-related infections [116,135]. The double-layer protective effect of BEVs, the presence of multiple functional molecules, and their regulatory ability to interact with the bacterial community have created significant difficulties in anti-biofilm treatment.

#### 4.5.1. Horizontal Transfer of Drug-Resistance Genes

BEVs are the important mediators that mediate the horizontal transfer of resistance genes and virulence genes within the biofilm, and they can also enhance the antibiotic tolerance of the biofilm through multiple pathways [116,136].

The lipid bilayer structure of BEVs can effectively protect nucleic acid substances such as plasmids, genomic DNA fragments, and sRNAs encapsulated inside through degradation by extracellular nucleases, enabling the long-distance and cross-species delivery of drug-resistance genes [111,113]. Studies have shown that BEVs secreted by *Acinetobacter baumannii* can mediate the horizontal transfer of carbapenem-resistance genes *blaOXA-24* and *blaNDM-1*, enabling previously sensitive strains to acquire drug resistance [116].

For example, the BEVs of *Pasteurella gallinarum* can completely transfer a drug-resistance island containing 11 non-plasmid drug-resistance genes to a homologous non-pathogenic strain, conferring the receptor bacteria with multiple drug-resistance phenotypes [135]. This gene transfer mode directly accelerates the evolution of drug resistance in the bacterial community and the formation of community-level drug-resistance phenotypes.

#### 4.5.2. Antibiotic Neutralization and Immune Evasion

BEVs can directly weaken the bactericidal efficacy of antibiotics through two core pathways. One is by delivering fully active antibiotic inactivators to directly degrade antibiotic molecules in the extracellular environment; the other is by exerting a “molecular decoy” effect through the membrane structure and drug-binding proteins, adsorbing and isolating antibiotics, reducing their direct contact with bacterial cells, and significantly reducing the effective drug concentration around the biofilm [116,135].

BEVs can synergistically enhance immune evasion with the biofilm matrix, blocking the synergistic effect of host immune clearance and treatment [116,136]. Pathogenic BEVs can compete for the binding of immune effectors such as complement and antibodies, reduce the probability of source bacteria being recognized, and assist biofilm bacteria in immune escape [65].

#### 4.5.3. High Heterogeneity Increases Therapeutic Difficulty

In addition, the high heterogeneity of BEVs significantly increases the difficulty of developing targeted therapeutic drugs and clinical translation. The size, surface markers, composition of contents, and biological functions of BEVs will dynamically change with strain type, environmental pressure, growth stage, and biofilm development stage, making it difficult for a single targeted drug to cover all BEV subtypes, and resulting in a narrow therapeutic window and significantly increased difficulty in clinical translation [109,135,137].

BEVs not only directly regulate the whole life cycle of biofilms, but also indirectly affect the development of biofilms by regulating the host immune response. Therefore, BEVs may be important factors mediating immune regulation and biomembrane regulation, and are an important mechanism for persistent infection. This will be elaborated in the next chapter.

## 5. The Bridging Role of BEVs in Immune Regulation and Biofilm Interaction

**Figure 3:**
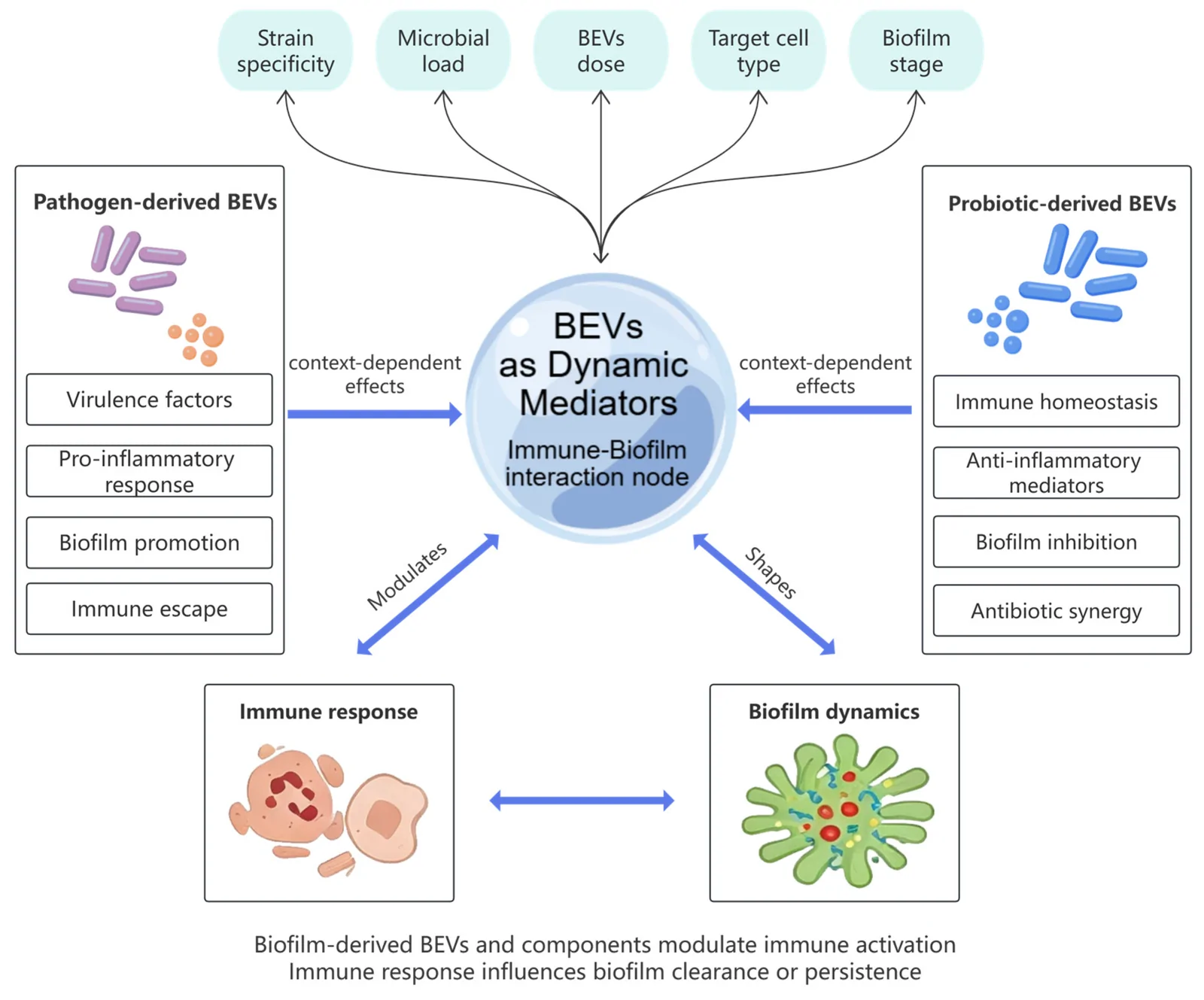
The bridging role of BEVs in immune regulation and biofilm interaction. Abbreviations: BEVs, bacterial extracellular vesicles.

BEVs play a crucial bridging role in the bidirectional pathways of “pathogen–host inflammation–biofilm formation” and “probiotics–immune regulation–biofilm deconstruction”. Therefore, BEVs are dynamic intervention nodes for microbial infection and biofilm regulation in the body (Figure 3).

### 5.1. Pathogenic BEVs: The Vicious Cycle of Infection–Inflammation–Biofilm

BEVs serve as a crucial medium for the interaction between the biofilm and the host immune system [65]. Certain pathogenic bacteria can promote the formation of biofilms through BEVs, protecting themselves from immune system attacks. Furthermore, pathogenic bacteria may stimulate the host immune system by releasing BEVs, competing for the binding of immune effectors, weakening the immune factor effect, reducing the probability of the source bacteria being recognized, and protecting the source bacteria, thereby achieving immune evasion [65]. During this process, BEVs act as mediators for the formation of biofilm and immune regulation. Simultaneously with the formation of biofilm, BEVs create a vicious cycle of immune suppression.

Studies have shown that *S. mutans* forms biofilms to adapt to the oral environment, and its BEVs can significantly promote the formation of biofilms [66]. Furthermore, these BEVs inhibit or disrupt the host’s immunity through multiple mechanisms, thereby creating a more favorable environment for the bacteria (including those in the biofilm) to survive [66]. *S. mutans* BEVs induce macrophages to switch to glycolysis (pro-inflammatory phenotype), leading to the release of many inflammatory factors, which instead causes tissue damage and exacerbates inflammatory damage rather than effectively eliminating the bacteria, facilitating the continuous infection of the bacteria. These BEV pretreatments can also significantly reduce the phagocytic ability of macrophages against bacteria. Additionally, BEVs can increase the attachment of bacteria to host cells, which is the first key step in the formation of a biofilm [66]. From this, bacteria may establish a feedback loop through BEVs, where immunosuppression promotes the formation of biofilms, and the biofilms in turn enhance the immunosuppressive cargo release of BEVs.

In the intestine, pathogen-derived BEVs collectively shape a local microenvironment characterized by increased nutrient availability, impaired physical barrier function, and disrupted immune surveillance. This altered microenvironment significantly reduces the difficulty of pathogen colonization and provides ideal conditions for bacterial aggregation and adhesion, which may also facilitate biofilm formation, although the specific role of pathogen-derived BEVs in intestinal biofilm formation remains incompletely understood [138].

Therefore, BEVs derived from pathogenic bacteria can directly promote the formation and maturation of biofilms by delivering virulence factors, nucleic acids, and quorum sensing signals, and can simultaneously regulate the host immune response (such as inducing low-level inflammation and inhibiting immune clearance), thereby assisting in the establishment of an immune escape ecosystem for the biofilm, forming a vicious cycle of “infection–inflammation–biofilm”.

### 5.2. Probiotic BEVs: Potential Intervention Nodes for Breaking the Cycle

Due to the dual capabilities of BEVs in suppressing immunity and promoting biofilms, they pose a significant risk to the health of the organism. BEVs from some probiotic strains may be an effective means to address bacterial biofilms in the body. Probiotic BEVs contain almost no toxic substances and can enhance immune function and inhibit excessive inflammation. Furthermore, some probiotic BEVs have anti-biofilm functions and can carry antibiotics or other drugs for a synergistic antibacterial effect.

For example, the BEVs produced by *E. coli* Nissle 1917 have the functions of regulating immunity and inhibiting inflammation [139]. Furthermore, the BEVs produced by *E. coli* Nissle 1917 can also carry tobramycin (TOB), demonstrating antibacterial and anti-biofilm capabilities. The antibacterial effect of these drug-loaded BEVs on bacteria within biofilms is 20,000 times stronger than that of free TOB [131].

The BEVs of *Lpb. plantarum* can inhibit inflammatory responses and promote M2 polarization of macrophages [140]. Moreover, these BEVs can also inhibit the biofilm of *L. monocytogenes* [126]. Other probiotics, such as *C. butyricum,* also produce BEVs, which have significant immunomodulatory effects. In the future, these probiotic BEVs can also be used as carriers for antibiotic research and in anti-biofilm applications [141].

Furthermore, these probiotic BEVs can promote the expression of tissue repair factors (such as transforming growth factor-β (TGF-β)), accelerate post-infection healing, and by inhibiting inflammation and enhancing immunity, may contribute to interrupting the vicious cycle of infection–inflammation–biofilm formation, exerting effective antibacterial effects [142].

Therefore, some probiotic BEVs show potential to modulate the “infection–inflammation–biofilm formation” cycle by delivering anti-inflammatory mediators, immune regulatory molecules and biofilm interference components. It is possible that these probiotic BEVs can not only alleviate pathological inflammation by regulating immune balance (such as enhancing the mucosal barrier and inducing regulatory immune responses), but also directly inhibit the formation of pathogenic bacteria biofilms or disrupt their structure. Therefore, BEVs may not only serve as functional mediators for the interaction between biofilms and the immune system, but also represent an important target and tool for intervening in infectious and inflammatory diseases.

## 6. Pathogenic Versus Probiotic-Derived BEVs: Similarities and Differences

BEVs from different bacterial sources share a common structural basis, but there are significant differences in molecular composition and biological functions. This chapter systematically compares the similarities and differences between pathogenic and probiotic-derived BEVs from the dimensions of structure, molecular composition and core function. The detailed mechanisms of immune regulation and biofilm regulation are described in Chapter 3 and Chapter 4 respectively.

### 6.1. The Similarity of BEVs Derived from Pathogenic Bacteria and Probiotic Bacteria

#### 6.1.1. Morphological and Structural Similarity

The BEVs derived from pathogenic bacteria and probiotic bacteria exhibit significant morphological similarity at the structural level. Transmission electron microscopy observations revealed that both BEVs derived from Gram-negative bacteria and BEVs derived from Gram-positive bacteria presented typical spherical or cup-shaped morphologies, with diameters mainly ranging from 20 to 400 nm [143,144,145]. This morphological feature is similar to that of exosomes derived from eukaryotic cells, reflecting the commonality of membrane vesicles at the biophysical level. For instance, the average diameter of BEVs released by the *Vibrio ordalii* is approximately 215 nm, which strongly overlaps with the size range of BEVs derived from probiotic bacteria [146]; and the diameter distribution of *Helicobacter pylori* BEVs under acidic and neutral culture conditions also falls within a similar range [147].

#### 6.1.2. Molecular Composition Similarity

The BEVs derived from pathogenic bacteria and probiotic bacteria have similarities in their molecular composition. Their composition generally includes membrane lipids, proteins, nucleic acids, and metabolites, etc. [143].

In terms of membrane lipids, phosphatidylethanolamine, phosphatidylglycerol, and cardiolipin are the main lipid components shared by BEVs from Gram-negative and Gram-positive bacteria. These lipids not only form the basic framework of the membrane but also participate in the membrane fusion between vesicles and host cells as well as signal recognition [148,149].

In the protein component, outer-membrane/membrane proteins, periplasmic/cytoplasmic proteins, and proteins related to the secretion system are all found in both types of BEVs, although the specific types and abundances vary [5,49,150,151].

Both different sources of BEVs contain nucleic acid components. For example, stress-induced BEVs of *Vibrio cholerae* can mediate the transfer of resistance genes [19]. Similarly, probiotic BEVs also carry functional RNA molecules. For instance, BEVs produced by engineered *Bacillus subtilis* and *Pseudomonas putida* containing double-stranded RNA can be transported across species to fungal cells and exert antifungal protective effects [152].

The loading of metabolites and small molecules is also a common feature of both types of BEVs. *Xanthomonas citri* BEVs are rich in iron carriers and essential metal ions and can serve as the sole carbon source to support bacterial growth [148], while BEVs derived from the *Faecalibacterium prausnitzii* carry small molecules such as phosphatidylcholine and can alleviate viral infections by regulating the metabolic balance of the intestinal microecology [153].

#### 6.1.3. Core Biological Function Similarity

The BEVs derived from pathogenic bacteria and probiotic bacteria exhibit significant similarities in core biological functions, mainly manifested in information transmission and environmental adaptation, etc. [143].

As information transmission carriers, BEVs can mediate long-distance communication between bacteria and between bacteria and the host, and can achieve the targeted delivery of functional molecules without direct cell contact [19,154,155].

Environmental adaptation and stress response are another important functional commonality of BEVs. Both types of BEVs are involved in the adaptation of bacteria to environmental pressures, including antibiotic exposure, nutrient limitation, temperature changes, and the response to host defense factors [5,19,151]. Under antibiotic stress, BEVs can protect the parental strain by encapsulating antibiotic-degrading enzymes, transmitting resistance genes, or acting as “traps” to adsorb antibiotics [135,156]. Under iron-limitation conditions, the BEVs of *Aeromonas hydrophila* accumulate iron-regulated transport proteins, which may be involved in iron acquisition competition [157]. These functional commonalities reflect that BEVs are universal environmental adaptation tools for bacteria.

### 6.2. The Differences Between Pathogenic Bacteria and Probiotic Bacteria-Derived BEVs

#### 6.2.1. Differences in Surface Molecular Patterns and Immune Recognition

Although the overall morphology is similar, the similarity in morphology does not imply complete identity. There are discernible differences in the fine structural characteristics between pathogenic bacteria and probiotic BEVs. The surface molecular patterns of pathogenic bacteria and probiotic BEVs generally differ. The composition of pathogen-associated molecular patterns (PAMPs) of BEVs from different bacterial species sources varies. This ultimately leads to differences in the recognition pathways of host cells for vesicles and the inflammatory output patterns [78].

The surface of pathogenic BEVs usually enriches PAMPs, such as LPS, lipopolysaccharides (LOS), flagellar proteins, and peptidoglycan fragments, which are potent activators of host pattern recognition receptors (PRRs) and induce strong innate immune responses [144]. Probiotic and Gram-positive bacteria-secreted BEVs mostly rely on lipoproteins and peptidoglycan to activate the TLR2 signaling axis, and tend to induce anti-inflammatory mediators such as IL-10 and TGF-β, leading to a biased immune tolerance host response [78,158].

#### 6.2.2. Differences in Molecular Composition

The differences in molecular composition are the material basis for the functional differences in BEVs between pathogenic bacteria and probiotics. These differences manifest at multiple levels, such as the proteome, lipome, nucleome, and metabolome [5,143,151].

Proteomics analysis is the main method for revealing the functional differences in BEVs. Pathogenic bacteria BEVs generally contain virulence-related proteins [146]. For example, *Porphyromonas gingivalis*, a typical periodontal pathogen, secretes BEVs that are enriched with various pathogenic protein components. These BEVs can activate the host inflammatory pathway and mediate the damage and destruction of periodontal soft tissues and alveolar bone [159]; *Helicobacter pylori*, a typical gastric pathogen, secretes BEVs that can enrich VacA cytotoxin, CagA oncogene protein, and urease, among other virulence-related molecules. The vesicles deliver these factors to gastric epithelial cells, thereby inducing mucosal inflammation damage, genomic abnormalities, and promoting the occurrence and development of gastric cancer [160]; the BEVs of *Pasteurella multocida* contain Apx toxin and protease [161].

In contrast, the protein profile of probiotic BEVs generally contains functional protective factors and immune regulatory molecules [162]. *E. coli* Nissle 1917 (EcN), as a typical probiotic, secretes BEVs that contain regulatory molecules for maintaining the stability of tight junctions. These molecules can maintain the expression of occludin and claudin-14 and stabilize the membrane localization of tight junction proteins, thereby alleviating the damage to the intestinal epithelial barrier caused by pathogenic *E. coli* [73,163]; *Faecalibacterium prausnitzii* secretes BEVs that contain multiple functional protein components, which can reshape the intestinal microbiota structure and regulate the overall metabolic level of the intestine, thereby alleviating the damage caused by intestinal viruses [153]. It is worth noting that the protein components and other functional components of the BEVs produced by the same probiotic under different environmental conditions may also undergo significant changes. For example, the BEVs produced by *Lactobacillus rhamnosus* GG under alkaline conditions have stronger biological activity [68].

#### 6.2.3. Differences in Biological Function Outcomes

The biological functions of BEVs derived from pathogenic bacteria and probiotic bacteria often differ. This difference is manifested in the direction and outcome of host interaction [143,164]. BEVs from pathogenic bacteria often promote the establishment of infection, maintain a chronic infection state, and cause tissue damage [164,165]. BEVs from probiotic bacteria often maintain host homeostasis, enhance barrier function, and promote immune tolerance [68,162].

The specific immune regulatory mechanisms and biofilm regulatory effects of the two types of BEVs are detailed in Chapter 3 and Chapter 4, respectively.

It should be emphasized that these functional differences are not absolute. Some probiotic BEVs may exhibit pro-inflammatory activity, while certain pathogenic BEVs may also induce immune tolerance [141,143,166]. The final functional outcome depends on multiple factors, such as strain type, dose, action site, and host physiological state.

## 7. Translational Applications and Outlook in Immune Regulation and Anti-Biofilm Strategies

BEVs, due to their nano-vesicle structure and diverse biological functions, hold promise for various medical applications. However, it is important to distinguish between demonstrated applications and speculative future prospects. Currently, the only BEVs-based product with widespread clinical approval is the meningococcal OMV vaccine (Bexsero^®^) [167]. Most other applications remain at the preclinical stage, with evidence primarily deriving from in vitro assays and animal models [168,169]. In this section, we summarize the current state of evidence for each application area and note the translational status.

### 7.1. Targeted Translational Applications

#### 7.1.1. Vaccine Development and Immune Adjuvants

BEVs naturally contain pathogen-associated molecular patterns (PAMPs), such as LPS and flagellin, which can strongly activate the Toll-like receptor (TLR) signaling pathway and trigger a robust immune response. Therefore, BEVs themselves can serve as vaccine adjuvants or antigen carriers [170]. Currently, the meningococcal BEVs vaccine (Bexsero^®^) is successfully applied in clinical practice, demonstrating the safety and efficacy of BEVs vaccines [167].

In the field of bacterial infection prevention, BEV-based vaccines have the potential to induce both humoral and cellular immune responses against pathogenic bacteria. The natural vesicle structure and PAMP components of BEVs give them inherent adjuvant activity, which can effectively activate antigen-presenting cells and promote antigen presentation without additional adjuvants [171]. Engineered BEVs can further enhance vaccine efficacy by displaying specific pathogenic antigens on the vesicle surface, and have shown protective effects against in cancer cells and activate the host’s anti-immune memory [10]. While there is currently no research exploring BEVs as antibacterial vaccines, their proven success as cancer vaccine candidates suggests that they may hold great potential for developing novel vaccines against drug-resistant pathogenic bacteria.

#### 7.1.2. Antibacterial and Anti-Biofilm Drug Delivery Carriers

The vesicular structure of BEVs enables them to serve as drug carriers, capable of carrying drugs for combination therapy [9].

Studies have confirmed that drug-loaded BEVs have significantly enhanced antibacterial and anti-biofilm effects compared with free antibiotics. BEVs produced by *E. coli* Nissle 1917 can carry TOB, demonstrating antibacterial and anti-biofilm capabilities. This drug-loaded BEV has a killing effect on bacteria within biofilms that is 20,000 times stronger than free TOB [131]. These BEVs, through genetic engineering methods, carry the glycosyl hydrolase PslG on their surface, which can disrupt the biofilm structure and work synergistically with antibiotics to produce antibacterial and anti-biofilm effects [131]. In addition, antibiotics can induce bacteria to produce BEVs that encapsulate antibiotic molecules, and these antibiotic-loaded BEVs can penetrate eukaryotic cells and kill intracellular pathogens [129,130], providing a new strategy for the treatment of intracellular persistent bacterial infections.

#### 7.1.3. Mucosal Inflammation and Immune Homeostasis Regulation

Probiotic-derived BEVs have shown great potential in the treatment of mucosal inflammatory diseases and immune homeostasis regulation. Unlike live probiotics, BEVs can cross the mucus layer more easily and directly interact with intestinal epithelial cells and immune cells, avoiding the potential risks of live bacterial bacteremia in sensitive populations [71,72].

Studies have shown that probiotic BEVs can alleviate intestinal inflammation through multiple pathways. *E. coli* Nissle 1917 BEVs can reduce the level of proinflammatory factors, reduce the activity of inflammatory enzymes, enhance the expression of intestinal barrier markers, and then inhibit the development of DSS-induced colitis in mice [83]. *B. fragilis* BEVs deliver PSA to induce regulatory T cell differentiation and increase IL-10 production, exerting therapeutic effects on experimental colitis in mice [80]. In addition to regulating immune balance, probiotic BEVs can also directly inhibit the formation of pathogenic biofilms or disrupt pre-formed biofilms [126]. This dual effect of immune regulation and biofilm interference makes probiotic BEVs a potential intervention tool to break the “infection–inflammation–biofilm” vicious cycle, with broad application prospects in the treatment of chronic infectious and inflammatory diseases of the mucosa.

### 7.2. Core Translational Challenges and Outlook

Although BEVs show exciting potential in the fields of immune regulation and anti-biofilm therapy, their industrial production and clinical translation face significant challenges.

The standardization and scale-up challenges in the production of BEVs are significant factors restricting their large-scale production. The large-scale production of BEVs faces obstacles such as low output, high heterogeneity, and high costs [172]. The output of BEVs produced by wild-type bacteria is usually limited, which increases the cost of large-scale industrial extraction. Therefore, at present, constructing high-yield BEV strains through genetic engineering is an important research direction. For example, knocking out genes such as *tolR* and *mlaE* can significantly enhance the production of BEVs in *E. coli* Nissle 1917, reaching 180 times the yield of the wild type [131]. Moreover, the optimization of cultivation conditions is also an important means to enhance the production of BEVs. Environmental stress and chemical stress are effective methods to increase the production of BEVs [172]. Besides increasing the concentration, standardizing the cultivation conditions and extraction methods and improving the consistency of the components and functions of BEVs are also important directions in research on BEVs [173].

The clinical application of BEVs must address safety issues, including immunotoxicity, clearance mechanisms, and long-term effects [173]. Many BEVs (especially those of pathogenic bacteria) are indicated to contain LPS, and they may also contain various toxic factors, which may trigger inflammatory responses. Therefore, research on the attenuation of such BEVs, as well as the screening of safe BEV-producing strains, are also key research directions in the future. Moreover, the phenomenon of BEVs accumulating in unintended organs is also a potential risk. Studies have found that exogenous BEVs can aggregate in non-target organs, such as the heart, liver, and lungs [174], which may cause potential damage. Therefore, the in vivo behavior of BEVs (such as biodistribution, metabolism, and immunogenicity) needs to be fully evaluated. Furthermore, how to improve the safety and targeting ability of BEVs is also an important research direction for the future.

Overall, BEVs represent a promising platform for immune regulation and anti-biofilm therapy. Further well-designed preclinical studies and standardized clinical trials are required to translate these preliminary findings into practical clinical applications. Future research should focus on elucidating the in vivo mechanisms of BEVs in the bidirectional interaction between immune regulation and biofilm dynamics, and promoting a standardized system construction in BEV research and industrial production, so as to accelerate the clinical translation process.

## 8. Conclusions

BEVs have become a key factor in regulating interactions between bacteria and between bacteria and the host, and they are of great significance in the pathogenesis of infectious diseases and medical research. This review systematically summarizes the current research evidence regarding the biogenesis mechanism, immune regulation, biofilm regulatory function, and application potential of BEVs. It also focuses on the bridging role that BEVs play in connecting the host immune response and the dynamic changes in bacterial biofilms.

Based on a comprehensive analysis of previous studies, we propose a unified conceptual framework: the biogenesis mechanism of BEVs directly determines the cargo composition of BEVs, and the functional diversity of BEVs, leading to different outcomes in host immune response and biofilm development. BEVs from pathogenic bacteria not only promote biofilm construction, but can also help bacteria escape immune attack, and induce inflammatory tissue damage, thus forming a self-reinforcing vicious cycle of “infection–inflammation–biofilm formation”, which is an important reason for chronic and recurrent infections. However, some BEVs derived from probiotics have shown dual beneficial effects: on the one hand, they maintain mucosal immune homeostasis, and on the other hand, they interfere with pathogenic biofilm formation. Therefore, this class of BEVs is expected to be a bidirectional intervention target to break the above pathological cycle.

Despite the rapid development of research in the field of BEVs, there are three key knowledge gaps in existing reviews that have not been adequately filled. First, the biogenesis of BEVs and their functional diversity have not been systematically described, making it difficult to interpret the different experimental results. Second, most studies treat immune regulation and biofilm regulation as two independent processes, ignoring the dynamic interaction between them and the important mediating role played by BEVs. Thirdly, there are widespread methodological limitations in the isolation, quantification and in vivo tracing of BEVs, which are often underestimated, seriously affecting the reproducibility of studies and the prospects for clinical translation. This review seeks to address these shortcomings by integrating mechanistic understandings in biogenesis, immunology, and biofilm biology and critically assessing methodological deficiencies that have constrained the field.

In the future, it is urgently needed to establish a standardized method system and carry out well-designed in vivo studies to verify the “immune–biofilm crosstalk” model and fully tap the application potential of BEVs. The conceptual framework presented here provides a comprehensive perspective for understanding the pathogenesis of chronic bacterial infections. It also lays a theoretical foundation for the development of a new generation of anti-infection and anti-biofilm strategies based on engineered or probiotic-derived BEVs.

## Figures and Tables

**Table 1:** Comparison of six different BEVs.

Type	Source Organism	Biogenesis Mechanism	Membrane Structure	Contains Cytoplasmic Content?	Main Cargo
OMVs	Gram−	Outer-membrane blebbing	Single outer membrane	NO	Outer-membrane proteins, LPS, periplasmic proteins, hydrophobic molecules
OIMVs	Gram−	Outer + inner-membrane blebbing	Double membrane	YES	DNA, RNA, cytoplasmic proteins, membrane proteins
EOMVs	Gram−	Explosive cell lysis	Single outer membrane	YES	DNA, RNA, cytoplasmic proteins, endolysins
EOIMVs	Gram−	Reassembly after explosive lysis	Double membrane (irregular)	YES	Abundant DNA, RNA, cytoplasmic proteins
CMVs	Gram+	Cytoplasmic membrane blebbing	Single membrane	YES	Cytoplasmic proteins, DNA, RNA, secreted proteins
ECMVs	Gram+	Bubbling cell death	Single membrane	YES	DNA, RNA, endolysins, metabolic enzymes

Note. The classification, biogenesis mechanisms and cargo characteristics are compiled and adapted from Toyofuku et al. [1].

## Data Availability

No new data were created or analyzed in this study. Data sharing is not applicable to this article.

## Abbreviations

The following abbreviations are used in this manuscript:

BDNF	Brain-derived neurotrophic factor
BEVs	Bacterial extracellular vesicles
CMVs	Cytoplasmic membrane vesicles
DSS	Dextran sulfate sodium
ECMVs	Explosive cytoplasmic membrane vesicles
eDNA	Extracellular DNA
EOIMVs	Explosive outer–inner-membrane vesicles
EOMVs	Explosive outer-membrane vesicles
EPS	Extracellular polymeric substances
EVs	Extracellular vesicles
HGT	Horizontal gene transfer
HMNQ	4-hydroxy-3-methyl-2-(2-non-enyl)-quinoline
MVs	Membrane vesicles
NLRs	Nucleotide-binding oligomerization domain-like receptors
OIMVs	Outer–inner-membrane vesicles
OM	Outer membrane
OMPs	Outer-membrane proteins
OMVs	Outer-membrane vesicles
PAMPs	Pathogen-associated molecular patterns
PGN	Peptidoglycan
PRRs	Pattern recognition receptors
QS	Quorum sensing
sRNAs	Small RNAs
TLR	Toll-like receptor
TOB	Tobramycin
